# Repetition of Prediabetes Enhances the Risk of Developing Diabetes

**DOI:** 10.1155/2019/4916546

**Published:** 2019-03-12

**Authors:** Yoshihiro Numata, Toshihide Ohya, Yasuhiko Nitta, Yumiko Yoshinaka, Azusa Shogakiuchi, Akihiro Toyota

**Affiliations:** ^1^Chugoku Rosai Hospital Research Center for the Promotion of Health and Employment Support, Japan Organization of Occupational Health and Safety, 1-5-1, Hirotagaya, Kure City, Hiroshima 737-0193, Japan; ^2^Department of Internal Medicine, Chugoku Rosai Hospital, 1-5-1, Hirotagaya, Kure City, Hiroshima 737-0193, Japan

## Abstract

We attempted to clarify the severity of the risk of diabetes mellitus (DM) in the individuals who repeatedly fulfill the criteria for prediabetes in both fasting plasma glucose (FPG) and hemoglobin A1c (HbA1c). The subjects were 2347 individuals who underwent annual health checkup at our hospital. They were classified as normal glucose tolerance or prediabetes as their yearly status of glucose tolerance for three years; furthermore, the individuals classified as prediabetes were subclassified into 3 groups. Among them, we focused the individuals who fulfilled the criteria for prediabetes in both FPG and HbA1c, and this group was named as PD3. Similarly, all subjects were categorized into 4 groups by the frequency of the status of PD3 during three years. Moreover, all subjects were categorized into 8 patterns when PD3 status was positive. Then, we surveyed the development of diabetes for 5 years, and the incidence rates (IRs) and the age- and sex-adjusted odds ratios (ORs) were obtained. A total of 188 subjects developed diabetes. The individuals in the group of PD3 showed the highest IR of DM (33.6%). The values of ORs were 11.5, 20.0, and 63.5 when the frequencies of PD3 were one, two, and three, respectively. In the group whose frequency of PD3 was two, the individuals who had repeated the status of PD3 twice then moved to the status other than PD3 showed smaller risk of DM than the others in the same group. In conclusion, individuals who fulfill the criteria for prediabetes in both FPG and HbA1c were at a high risk of developing DM, and the risk was enhanced by repeating this status. On the other hand, changing the status from PD3 to others might reduce the risk of DM.

## 1. Introduction

The worldwide prevalence of diabetes mellitus (DM) is increasing; therefore, the importance of preventing DM is emphasized [1, 2]. A proper diet and sufficient exercise should be naturally introduced in the treatment of DM; on the other hand, such interventions are also available for preventing the development of DM [3, 4]. Type 2 DM gradually deteriorates an individual's glucose tolerance [5]. We believe that these interventions should be applied at the early stage of glucose tolerance deterioration to efficaciously treat DM. Prediabetes (PD) is the high risk status to develop DM [6, 7] and can be simply diagnosed by clinical data such as fasting plasma glucose (FPG) or hemoglobin A1c (HbA1c) [8]. We suggest that applying these interventions to all patients with PD is one of the strategies for preventing DM; however, the annual incidence rate of DM from PD was reported to be 5%-10%, and 5%-10% patients with PD could return to normal glucose tolerance (NGT) [6, 7]. We should understand that all patients with PD do not have similar risks of developing DM. Some reports showed the values of FPG and HbA1c could predict the development of DM, and these values were similar to the threshold values of the criteria for PD [9–11]. Recently, Heianza et al. showed that the individuals who fulfilled the criteria for PD in both FPG and HbA1c had the severe risk to develop DM among patients with PD [12]. Applying the interventions to the individuals in such status may be efficacious and cost-effective for preventing DM. Meanwhile, we know some individuals repeat such status at the time of annual health checkup. We conceive they are likely to develop DM with a high incidence and seriously need to have the interventions to prevent DM. However, any information about the severity of the risk has never been shown in such case. Therefore, this study is aimed at clarifying the severity of the risk of DM in the individuals who repeated the abnormal results in both FPG and HbA1c.

## 2. Methods

### 2.1. Study Participants and Diagnosis

This study has been approved by the ethics committee of Chugoku Rosai Hospital (Hiroshima, Japan). All participants were informed that their clinical data would be used for analysis and that they had the option to withdraw their consent at any time.

We collected and reviewed the records of annual health checkup of 3193 individuals who were conducted at Chugoku Rosai Hospital without missing in 2009, 2010, and 2011 (entry period). Most of the examinees were healthy employees of nearby companies around our hospital. We excluded 451 individuals who could not be followed up for five years (from 2012 to 2016, survey period). Furthermore, 304 individuals were excluded due to existing DM or a new diagnosis of DM by examination results upon entry into the study. Only one individual withdrew the consent to participate in this study and thus was excluded. Moreover, we attempted to find the individuals who had the disease or the medication affecting the examination results of FPG and HbA1c [13, 14]. After checking 2437 medical records carefully, we excluded 83 individuals due to iron deficiency anemia, 1 individual due to renal anemia, 1 individual due to autoimmune hemolytic anemia, 1 individual due to the past pancreatectomy, and 4 individuals due to the steroid therapy. The final study population comprised 2347 subjects.

In the entry period, each participant was classified as PD or NGT according to our criteria (shown in Table 1) which followed the criteria of American Diabetes Association [8]. In this study, we could not obtain results of oral glucose tolerance test (OGTT), and therefore, a diagnosis was made using the values of FPG and HbA1c. Furthermore, we categorized the subjects with PD into three groups. The first group was comprised of the individuals fulfilling the criteria of PD only in FPG, and this group was named as PD1. The second group was comprised of the individuals fulfilling the criteria only in HbA1c (PD2), and the third group was comprised of the individuals fulfilling the criteria in both of them (PD3). Then we reviewed their records of health checkup in the survey period, and annual status of glucose tolerance was diagnosed as DM, PD (PD1, PD2, and PD3), or NGT by yearly results of FPG and HbA1c. For diagnosis of DM in the survey period, we verified a subject's self-report of new diagnosis of DM or that the laboratory results were consistent with the criteria for diagnosing DM. The final outcome of this study was the development of DM. Once a subject was diagnosed as DM, the annual status of glucose tolerance has been DM since then.

### 2.2. Anthropometry and Examination Data

Height and weight were measured while a participant wore a hospital's uniform for health checkup. Body mass index (BMI) was calculated according to the established procedure: BMI = body weight(kg)/[body height(m)]^2^. All of the subjects completed proper overnight fasting, and blood samples were taken from their antecubital vein. Blood samples were immediately measured in the hospital's laboratory which is accredited by Japan Medical Association. The values of HbA1c as defined by the Japan Diabetes Society (JDS) in 2009 were converted to those of the National Glycohemoglobin Standardization Program (NGSP) according to the former report [15]. Our hospital has adopted the NGSP scale to express HbA1c values since then.

### 2.3. Statistical Analyses

Statistical analyses were performed using JMP12 (SAS Institute Japan, Tokyo, Japan). Differences in group characteristics were compared by the chi-square test for categorical variables. The one-way ANOVA and Tukey-Kramer test were used to compare differences of continuous variables. The age- and sex-adjusted odds ratios (ORs) of developing DM were obtained by logistic regression analyses for the identification of the severity of the risk in the groups of NGT, PD1, PD2, and PD3. Similarly, all subjects were categorized by the frequency of PD3 status during the entry period, and then the age- and sex-adjusted ORs of DM were calculated in these groups. Moreover, all subjects were categorized into eight patterns when the status of PD3 was positive during the entry period, and then the age- and sex-adjusted ORs of DM during the survey period were obtained by logistic regression analyses. *P* value of less than 0.05 was considered to be significant.

## 3. Results

### 3.1. Development of DM during the Survey Period

Firstly, we surveyed the association between the status of glucose tolerance in the first year (2009) and the outcome during the survey period. In 2009, 1014 individuals were classified as PD and 1333 as NGT. Furthermore, we categorized individuals with PD into three subgroups (Table 2). The numbers of individuals categorized as PD1, PD2, and PD3 were 507, 200, and 307, respectively. 188 individuals newly developed DM during the survey period. Of the new DM cases, 103 individuals (54.8%) were classified as PD3. Also, 33 individuals (17.6%), 38 individuals (20.2%), and 14 individuals (7.4%) were classified as PD2, PD1, and NGT, respectively. Their incidence rates (IRs) during the survey period were 33.6%, 16.5%, 7.5%, and 1.1%, respectively. The ORs of developing DM in the groups of PD3, PD2, and PD1 were, respectively, 45.5, 17.0, and 7.8 when compared with NGT.

We concluded the individuals classified as PD3 had the highest risk of developing DM among three subcategories of PD. Next, we examined the association between the repetition of this status (PD3) and the incidence of DM.  Table 3 shows that the incidence rate of DM increased in proportion to the frequency of PD3 during the entry period. The OR of developing DM in the group whose frequency of PD3 was one (named as Frequency-1) was 11.5 when compared with the group where the individuals had never undergone the status of PD3 (Frequency-0). Similarly, the ORs in the groups whose frequency was two (Frequency-2) and three (Frequency-3) were, respectively, 20.0 and 63.5.

### 3.2. Features of the Groups Categorized by the Frequency of PD3

In order to clarify the features of the groups of Frequency-1, Frequency-2, and Frequency-3, we performed statistical analyses about anthropometry and clinical factors which were reported to concern glucose tolerance [16–18].  Table 4 shows the mean values and standard deviations of them. The values of FPG and HbA1c significantly increased in proportion to the increase of the frequency of PD3 during the entry period. The mean values of age, systolic blood pressure (BP), diastolic BP, and fasting serum insulin (IRI) in the group of Frequency-3 were significantly higher than those of Frequency-1. On the other hand, the mean value of HDL cholesterol (HDL-C) was significantly lower than that of Frequency-1.

### 3.3. Effects of the Style When PD3 Is Positive

The group of Frequency-3 showed the highest risk of developing DM. However, the groups of Frequency-1 and Frequency-2 also showed severe risk of DM when compared with the group consisting of PD2, PD1, and NGT (Frequency-0). We analyzed the severity of the risk in eight patterns when PD3 was positive during the entry period (Table 5). In the group of Frequency-1, the individuals who were PD3-positive only in 2009 showed the relatively small values in IR and OR when compared with those who were positive only in 2010 or 2011. There seemed to be no difference in the severity of the risk between 2010 and 2011.

In the group of Frequency-2, the individuals who were PD3-positive in 2009 and 2010 but negative in 2011 apparently showed the small risk. Their IR and OR of DM during the survey period were 11.5% and 10.3, respectively. These values were almost half of the values of the other individuals in the same group and comparable to those in the group of Frequency-1. The individuals who were PD3-positive in 2010 and 2011 had the rather large risk of DM when compared with the individuals who were PD3-positive in 2009 and 2011.

## 4. Discussion

In the present study, a risk of developing DM was estimated using the values of FPG and HbA1c. Therefore, the risk might be erroneous when an individual had comorbidity or medication which affected the examination results of FPG and HbA1c. Iron deficiency anemia, hemolytic anemia, and renal anemia affect the value of HbA1c because the red blood cell turnover is not normal [14]. In case of iron deficiency anemia, the value of HbA1c may be elevated in an untreated patient but decreased in a patient getting better by medication, although the blood glucose level is normal. However, we could only have annual data and a little information about therapy in many cases. For this reason, we never excluded an individual with a diagnosis of anemia when the value of hemoglobin was within our normal range. Eventually, we excluded 85 individuals whose hemoglobin levels were below the lower limit of normal (Table 6). Moreover, an individual who had comorbidity directly affecting the value of FPG should be excluded. In our database for this study, we found 4 individuals having steroid therapy and 1 individual with past partial pancreatectomy. Medication with steroid causes increasing a blood glucose level [14]. 3 individuals were administered 5-10 mg of prednisolone for treatment. We could not know the dose of prednisolone for the patient with IgA retinopathy because she had medication at another hospital. We excluded these 4 individuals owing to steroid therapy. Pancreatectomy would result in insulin insufficiency and deterioration of glucose tolerance. Similarly, we excluded her. We could never find other comorbidities affecting the values of FPG and HbA1c [14]. In total, we excluded 90 individuals shown in Table 6. In the excluded individuals, the value of IR of DM was 16.7%, and it was twice as many as that of this study. Leaving them as subjects might have made our conclusions obscure.

PD is the status indicating relatively high risk of the future development of DM [6, 7]. In the present study, we categorized individuals with PD into three groups following the former report [12]. We hypothesized that the risk of developing DM would increase in the order of NGT, PD1, PD2, and PD3 and regarded them as the classification of risk. In our opinion, the reasons why individuals in the PD3 group have a high risk of developing DM are as follows. Individuals in the group of PD1 have occasional hyperglycemia, and its frequency is not so high; thus, the level of HbA1c is still normal. In the group of PD2, although hyperglycemia occurs frequently, there is a potential that hyperglycemia can return to the normal range after overnight fasting. In the group of PD3, overnight fasting cannot bring hyperglycemia to the normal range, and PD3 implies the worst glucose tolerance among three. The IRs and the ORs of DM during the survey period were identical to our hypothesis, and more than half of the new DM patients belonged to the PD3 group in 2009. Our results slightly differ from those of Heianza et al. [12], but the different cohort could account for it. Our results confirm the important thing that an individual who fulfills the criteria for PD in both FPG and HbA1c has a severe risk of developing DM. In addition, 21.7% of the individuals in the group of PD1 could return to NGT; however, only 2.9% of those in the groups of PD3 could return to NGT. Without treatments, the individuals classified as PD3 could hardly return to NGT and either retained their high risk status or developed DM, which also suggests that there is a severe risk of DM in the group of PD3.

We know some individuals repeat the status of PD3. Therefore, we examined whether such individuals had severer risk of DM (Table 3). The IRs and ORs of DM increased in proportion to the frequency of PD3, which suggests repetition of PD3 status would enhance the risk of developing DM. Furthermore, we could find more information from this table. The group of Frequency-0 was comprised of the individuals who had never undergone PD3 status during the entry period. The status of each individual in this group was PD2, PD1, or NGT. However, the IR of DM in this group was lower than that of the IRs of PD2 and PD1 and similar to NGT in 2009 (shown in Table 2). It suggests that most of the new DM cases would go through PD3 status to develop DM. According to our hypothetical classification of the risk of DM, we surveyed the association between the incidence of DM and the worst status during the entry period in the Frequency-0 group. 10 (3.5%) of 290 individuals at PD2, 8 (1.1%) of 701 individuals at PD1, and 1 (0.2%) of 626 individuals at NGT, respectively, developed DM. Therefore, we consider an individual who remains the status other than PD3 has a relatively small risk of DM.

Focusing the features of the groups of Frequency-0, Frequency-1, Frequency-2, and Frequency-3, we could find the significant difference in the values of FPG and HbA1c (Table 4). The mean values of them increased in proportion to the frequency of PD3, which suggests glucose tolerance becomes worse in this order. It also supported our results mentioned above: the more the frequency of PD3 was, the severer the risk of DM became. Also, we found the significant difference in the mean values of age, systolic BP, diastolic BP, IRI, HDL-C, and TG between the groups of Frequency-1 and Frequency-3. Including the mean values of BMI, AST, and ALT, all of these values became worse in the order of PD1, PD2, and PD3. These data might reflect the deterioration of their glucose tolerance; however, the small differences resulted in finding the significances only between Frequency-1 and Frequency-3.

We found interesting possibility in the results shown in Table 5. The risk of DM in the group of Frequency-2 was severer when compared with the group of Frequency-1. However, the individuals who were PD3-positive in 2009 and 2010 but negative in 2011 showed smaller risk than the other individuals in the group of Frequency-2, and the risk was similar to that of Frequency-1. We think having the status other than PD3 in 2011 might reduce their risks. Also, we think that these results indicate the risk of DM in the status of PD3 could become small by moving to and keeping other status even if an individual is in the high risk status now.

In conclusion, the present study confirms that individuals in the status fulfilling the criteria for PD in both FPG and HbA1c have the high risk of DM progression and shows that repetition of this status will enhance the risk of DM. Furthermore, many of new DM cases are considered to go through PD3 status to develop DM, but the risk of DM is not so severe when an individual stays the status other than PD3. Moreover, changing the status from PD3 to others by intervention may reduce the risk of DM. In order to ensure our conclusions, similar studies in a large cohort and in a different ethnic group will be necessary, and prospective studies will also be needed to examine whether intervention to this status can actually reduce the risk of DM. Nevertheless, we recommend that an individual in the status of PD3, especially repeating this status at the time of annual health checkup, should be proactively treated to prevent DM.

## Figures and Tables

**Table 1:** Diagnostic criteria in this study.

*Normal glucose tolerance (NGT)*
FPG < 100 mg/dL and HbA1c < 5.7%
*Prediabetes (PD)*
FPG: 100 mg/dL to 125 mg/dL or HbA1c: 5.7% to 6.4%
*Diabetes mellitus (DM)*
FPG ≥ 126 mg/dL or HbA1c ≥ 6.5%

FPG: fasting plasma glucose; HbA1c: hemoglobin A1c.

**Table 2:** Risk of developing diabetes in prediabetes.

	*n*	Outcome					IR (%)	OR	*P* value
		NGT	PD1	PD2	PD3	DM			
NGT	1333	687	162	332	138	14	1.1	1	
PD1	507	110	150	56	153	38	7.5	7.8	<0.0001
PD2	200	15	3	92	57	33	16.5	17.0	<0.0001
PD3	307	9	8	42	145	103	33.6	45.5	<0.0001
Entire	2347	821	323	522	493	188	8.2		

NGT: normal glucose tolerance; PD1, PD2, and PD3: subgroups of prediabetes; DM: diabetes mellitus; IR: incidence rate of DM; OR: odds ratio of DM. *P* value shows the result of the likelihood ratio test in logistic regression analysis.

**Table 3:** Development of diabetes and the frequency of PD3 status.

Frequency	*n*	DM	IR (%)	OR	*P* value
0	1617	19	1.2	1	
1	322	40	12.4	11.5	<0.0001
2	208	41	19.7	20.0	<0.0001
3	200	88	44.0	63.5	<0.0001

DM: diabetes mellitus; IR: incidence rate of DM; OR: odds ratio of DM. *P* value shows the result of the likelihood ratio test in logistic regression analysis.

**Table 4:** Features of the groups classified by the frequency of PD3 status.

Frequency	0	1	2	3	SD
Male : female	1019 : 598	203 : 119	149 : 59	147 : 53	
Age	47.5 ± 9.3	51.7 ± 8.8	52.8 ± 8.2	54.7 ± 8.0	c
BMI	22.5 ± 3.1	23.8 ± 3.1	24.0 ± 3.6	24.3 ± 3.7	
Systolic BP (mmHg)	118.8 ± 16.3	123.5 ± 16.6	124.3 ± 16.3	127.8 ± 17.8	c
Diastolic BP (mmHg)	74.1 ± 10.7	76.6 ± 10.6	77.6 ± 10.6	79.6 ± 11.2	c
FPG (mg/dL)	94.1 ± 7.5	99.1 ± 6.9	103.1 ± 7.2	109.9 ± 6.3	a, b, c
HbA1c (%)	5.31 ± 0.24	5.59 ± 0.19	5.67 ± 0.19	5.88 ± 0.15	a, b, c
IRI (*μ*U/mL)	5.62 ± 2.82	6.59 ± 3.40	6.66 ± 3.07	7.54 ± 4.20	c
LDL-C (mg/dL)	118.9 ± 28.5	133.6 ± 29.3	129.4 ± 28.4	136.6 ± 30.1	
HDL-C (mg/dL)	67.0 ± 16.1	63.5 ± 15.9	63.0 ± 17.1	59.0 ± 14.2	c
TG (mg/dL)	102.6 ± 82.0	120.8 ± 78.9	127.6 ± 85.8	139.7 ± 106.5	
AST (IU/L)	21.4 ± 8.9	22.4 ± 8.4	22.8 ± 9.1	24.3 ± 9.9	
ALT (IU/L)	22.3 ± 14.6	25.4 ± 16.4	26.7 ± 19.6	29.0 ± 19.2	
*γ*-GTP (IU/L)	37.8 ± 43.7	37.8 ± 30.7	45.9 ± 53.2	45.8 ± 36.7	

SD: significant difference; BP: blood pressure; FPG: fasting plasma glucose; HbA1c: hemoglobin A1c; IRI: fasting serum insulin; LDL-C: LDL cholesterol; HDL-C: HDL cholesterol; TG: triglyceride. Except gender, values in this table mean average ± standard deviation. The row of “SD” shows the significant difference among the groups; “a” means the significant difference between frequencies 1 and 2, “b” means 2 and 3, and “c” means 1 and 3.

**Table 5:** Risk of diabetes and the positive style of PD3 status.

2009	2010	2011	*n*	DM	IR (%)	OR	*P* value
—	—	—	1617	19	1.2	1	
PD3	—	—	31	3	9.7	8.8	0.0009
—	PD3	—	66	9	13.6	12.5	<0.0001
—	—	PD3	225	28	12.4	11.5	<0.0001
PD3	PD3	—	26	3	11.5	10.3	0.0004
—	PD3	PD3	132	29	22.0	22.9	<0.0001
PD3	—	PD3	50	9	18.0	17.9	<0.0001
PD3	PD3	PD3	200	88	44.0	62.8	<0.0001

DM: diabetes mellitus; IR: incidence rate of DM; OR: odds ratio of DM. The rows of 2009, 2010, and 2011 show the yearly status of glucose tolerance; “—” means the status other than PD3 (NGT, PD1, and PD2). *P* value shows the result of the likelihood ratio test in logistic regression analysis.

**Table 6:** Comorbidities of the excluded subjects.

(1) Anemia	*n*	Hb (g/dL)	FPG (mg/dL)	HbA1c (%)	PD3	DM
Iron deficiency (M)	12	11.2-13.8	83-105	4.7-5.9	3	2
Iron deficiency (F)	71	9.7-11.3	70-122	4.4-6.1	8	11
Hemolytic (M)	1	12.1-13.3	103-118	5.6-5.8	1	0
Renal (M)	1	10.9-12.0	85-99	5.6-5.8	0	0

(2) Others	*n*	Medication	FPG (mg/dL)	HbA1c (%)	PD3	DM
SLE (F)	2	PSL 5 mg	92-98	5.5-5.8	1	1
IgA retinopathy (F)	1	PSL^∗^	102-113	5.9-6.1	1	0
HES (M)	1	PSL 10 mg	96-101	5.1-5.2	0	0
Pancreatectomy (F)	1		92-109	5.9-6.1	1	1

^∗^Dose unknown. Hb: hemoglobin; FPG: fasting plasma glucose; PD3: subclassification of prediabetes in this study; DM: diabetes mellitus; (M): male; (F): female; SLE: systemic lupus erythematosus; HES: hypereosinophilic syndrome; PSL: prednisolone. Values of Hb, FPG, and HbA1c imply the lowest datum (average of the lowest data in the case of multiple) to the highest datum (average of the highest data in the case of multiple) during the entry period.

## Data Availability

The data used to support the findings of this study have not been made available because the original data were personal results of annual health checkups. The participants in this study have never consented to disclose their results.
